# Supplementary material for: Distribution and risk assessment of pesticide residues in sediment samples from river Ganga, India
Source: PLoS One. 2023 Feb 2;18(2):e0279993. doi: 10.1371/journal.pone.0279993 (PMC9894440; doi:10.1371/journal.pone.0279993)
Supplement: S1 Table — (PDF) [file pone.0279993.s001.pdf]

Table S1: Limit of detection (LOD), limit of quantification (LOQ) and recovery percentage of sediments fortified with 10 µg/kg (n=10) of pesticides in sediment.

Pesticide	LOD(µg/kg)	LOQ(µg/ kg)	% recovery
Chlordane	0.156	0.46	82
Methoxychlor	0.191	0.58	87
Dichlorvos	0.173	0.56	77
Malathion	0.087	0.275	90
Heptachlor	0.093	0.289	76
Cypermethrin	0.141	0.429	90
Azinphosmethyl	0.094	0.291	85
Tridemorph	0.095	0.295	83
Dimethoate	0.087	0.267	77
Atrazine	0.099	0.297	79
Binapacryl	0.148	0.45	89
Nuarimol	0.086	0.265	73
Methyl parathion	0.091	0.285	78
