# Supplementary material for: Distribution and risk assessment of pesticide residues in sediment samples from river Ganga, India
Source: PLoS One. 2023 Feb 2;18(2):e0279993. doi: 10.1371/journal.pone.0279993 (PMC9894440; doi:10.1371/journal.pone.0279993)
Supplement: S2 Table — (PDF) [file pone.0279993.s002.pdf]

Table S2: Few of the international studies showing concentration of observed pesticides.

Heptachlor	1.75 µg/kg dw	Unyimadu *et al*., 2019
Heptachlor	0.088 µg/kg dw	Kafilzadeh *et al*., 2015
Cypermethrin	2.65 µg/g dw	Phillips *et al*., 2010
Heptachlor	0.625 µg/kg dw	Ogbeide *et al*., 2019
Malathion	0.08 ng/g dw	Ccanccapa *et al*., 2016
Chlordane	1.54 ng/g dw	Yang *et al*., 2015
Methoxychlor	3.94 ng/g dw	Yang *et al*., 2015
Heptachlor	7.69 ng/g dw	Zhou *et al*., 2006
Heptachlor	0.36 µg/g dw	Olutona et al., 2014
Chlordane	1.35 µg/g dw	Olutona et al., 2014
Methoxychlor	0.51 µg/g dw	Olutona et al., 2014
Chlordane	0.074 µg/kg dw	Kafilzadeh *et al*., 2012
Heptachlor	0.081 µg/kg dw	Kafilzadeh *et al*., 2012
Dimethoate	0.0346 µg/kg dw	Tokatli, 2020
Heptachlor	3.51 ng/g dw	Syed *et al*., 2014
