# Supplementary material for: Distribution and risk assessment of pesticide residues in sediment samples from river Ganga, India
Source: PLoS One. 2023 Feb 2;18(2):e0279993. doi: 10.1371/journal.pone.0279993 (PMC9894440; doi:10.1371/journal.pone.0279993)
Supplement: S3 Table — (PDF) [file pone.0279993.s003.pdf]

Table S3: Represents the estimated input parameters used in calculation of chronic daily intake (USEPA, 2017).

Parameter	Unit	Min	Mean	Max
Ingestion rate	mg d^{-1}	0.1	50	25
Exposure Frequency	d yr^{-1}	180	345	365
Exposure duration	yr	11.36	-	-
Body weight adult	kg	76.71	-	-
Body weight children	kg	6.5	15	26.1
Average life span adult	d	8760	-	-
Average life span child	d	2190	-	-
Surface area	cm^{-2} d^{-1}	760	1.530	4.220
Inhalation rate	m^{-3} d^{-1}	2.4	26.8	17.7
Particle emission factor	m^{-3} kg^{-1}	1.36E09	-	-
Dermal absorption factor	-	0.13	-	-
Dermal surface factor	mg cm^{-1}	0.65	-	-

- = NA

IR = Ingestion rate, CF = Conversion factor, EF = Exposure frequency, ED = Exposure duration, BW = Body weight, AT = Average life span, PEF = Particle emission factor, IAR = Inhalation rate, SA = Surface area, ABS = Dermal absorption factor, AF = Dermal surface factor.
