# Supplementary material for: Distribution and risk assessment of pesticide residues in sediment samples from river Ganga, India
Source: PLoS One. 2023 Feb 2;18(2):e0279993. doi: 10.1371/journal.pone.0279993 (PMC9894440; doi:10.1371/journal.pone.0279993)
Supplement: S4 Table — (PDF) [file pone.0279993.s004.pdf]

Table S4: Input values of oral reference dose and cancer slope factor of each pesticide (USEPA, 2017).

Pesticide	Reference dose	Slope factor
Chlordane	0.00006	0.35
Dimethoate	0.0002	-
Malathion	0.02	0.0038
Atrazine	0.035	0.22
Heptachlor	0.0005	4.5
Dichlorvos	0.0005	0.29
Azinphosmethyl	0.2	-
Cypermethrin	0.01	-
Methoxychlor	0.005	-
Methyl parathion	0.00025	-
Ethylthiourea	0.00008	-
Nuarimol	0.006	-
Chlormequat	0.09	-
Tridemorph	0.010	-

- = NA
