# Supplementary figures and images for: Distribution and risk assessment of pesticide residues in sediment samples from river Ganga, India
Source: PLoS One. 2023 Feb 2;18(2):e0279993. doi: 10.1371/journal.pone.0279993 (PMC9894440; doi:10.1371/journal.pone.0279993)

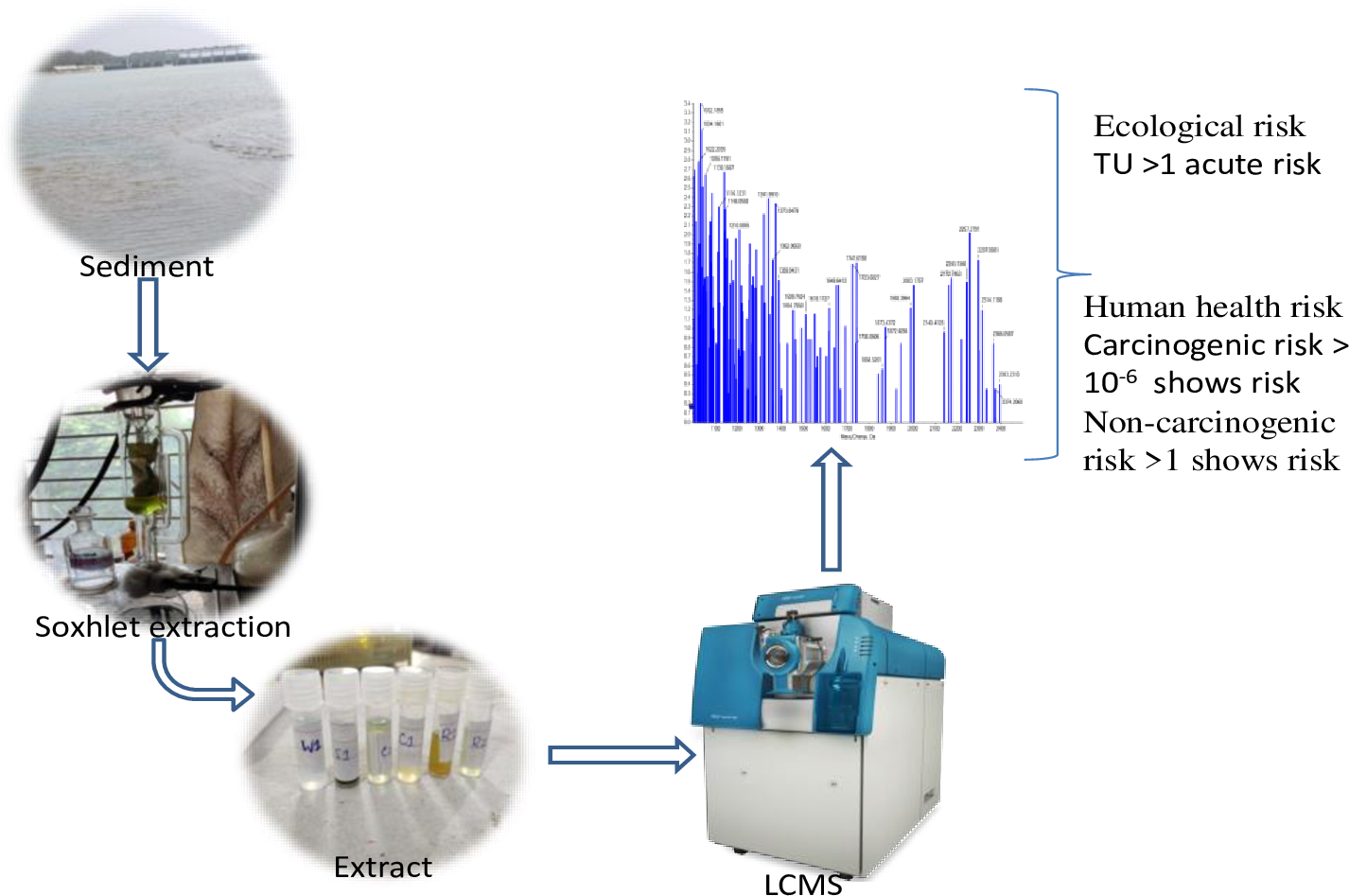

Supplement: S1 Graphical abstract — (TIF) [file pone.0279993.s005.tif]
