# Supplementary material for: Distribution and risk assessment of pesticide residues in sediment samples from river Ganga, India
Source: PLoS One. 2023 Feb 2;18(2):e0279993. doi: 10.1371/journal.pone.0279993 (PMC9894440; doi:10.1371/journal.pone.0279993)
Supplement: Attachment — Submitted filename: PONE-D-22-19870_reviewer.pdf [file pone.0279993.s007.pdf]

PLOS ONE

Distribution and risk assessment of pesticide residues in sediment samples from river Ganga, India

--Manuscript Draft--

Manuscript Number:	PONE-D-22-19870
Article Type:	Research Article
Full Title:	Distribution and risk assessment of pesticide residues in sediment samples from river Ganga, India
Short Title:	Distribution and risk assessment of pesticide residues in sediment samples from river Ganga, India
Corresponding Author:	ZESHAN Umar SHAH<br>Aligarh Muslim University Faculty of Life Sciences<br>Aligarh, UP INDIA
Keywords:	River Ganga; Pesticides; sediments; Ecological risk; Human health risk
Abstract:	Indiscriminate use of pesticides leads to their entry in to the bottom sediments, where they are absorbed in the sediment's particle and thus, may become the consistent source of aquatic pollution. The present work was carried out to evaluate pesticide residues in the sediment samples and associated human health risk of commonly used pesticides along the basin of river Ganga. Total of 16 pesticides were analyzed along three stretches of river Ganga. The concentration of pesticides in the upper stretch ranged from ND to 0.103 µg/kg, in the middle stretch ND to 0.112 µg/kg, and in the lower stretch ND to 0.105 µg/kg. Strong positive correlation was found between total organic carbon and total pesticide residues in sediment samples. Carcinogenic and non-carcinogenic values were estimated below the threshold limit suggesting no associated risk. Risks associated with the inhalation route of exposure were found to be higher than the dermal and ingestion routes. Children were found at higher risk at each site from multiple routes of exposure than adult population groups. Toxic unit values were found to be below the threshold value suggesting no risk associated with exposure of pesticides from sediments. However, long term effects on ecological quality due to consistent pesticide exposure must not be ignored. Therefore, the present study focuses on concrete efforts to reduce the threat of pesticide pollution in the river system and possible human health risk.
Order of Authors:	ZESHAN Umar SHAH
	ZESHAN SHAH, Associate Professor
Additional Information:	
Question	Response
**Financial Disclosure**<br><br>Enter a financial disclosure statement that describes the sources of funding for the work included in this submission. Review the submission guidelines for detailed requirements. View published research articles from *PLOS ONE* for specific examples.<br><br>This statement is required for submission and **will appear in the published article** if the submission is accepted. Please make sure it is accurate.	No funding available to mention

Powered by Editorial Manager® and ProduXion Manager® from Aries Systems Corporation

**Unfunded studies**<br>Enter: *The author(s) received no specific funding for this work.*<br><br>**Funded studies**<br>Enter a statement with the following details:<br>• Initials of the authors who received each award<br>• Grant numbers awarded to each author<br>• The full name of each funder<br>• URL of each funder website<br>• Did the sponsors or funders play any role in the study design, data collection and analysis, decision to publish, or preparation of the manuscript?<br>• **NO** - Include this sentence at the end of your statement: *The funders had no role in study design, data collection and analysis, decision to publish, or preparation of the manuscript.*<br>• **YES** - Specify the role(s) played.<br><br>* typeset	
**Competing Interests**<br><br>Use the instructions below to enter a competing interest statement for this submission. On behalf of all authors, disclose any competing interests that could be perceived to bias this work—acknowledging all financial support and any other relevant financial or non-financial competing interests.<br><br>This statement is required for submission and **will appear in the published article** if the submission is accepted. Please make sure it is accurate and that any funding sources listed in your Funding Information later in the submission form are also declared in your Financial Disclosure statement.<br><br>View published research articles from *PLOS ONE* for specific examples.	No authors have competing interest

**NO authors have competing interests**<br><br>Enter: *The authors have declared that no competing interests exist.*<br><br>**Authors with competing interests**<br><br>Enter competing interest details beginning with this statement:<br><br>*I have read the journal's policy and the authors of this manuscript have the following competing interests: [insert competing interests here]*<br><br>* typeset	
**Ethics Statement**<br><br>Enter an ethics statement for this submission. This statement is required if the study involved:<br><br>• Human participants<br>• Human specimens or tissue<br>• Vertebrate animals or cephalopods<br>• Vertebrate embryos or tissues<br>• Field research<br><br>Write "N/A" if the submission does not require an ethics statement.<br><br>General guidance is provided below. Consult the submission guidelines for detailed instructions. **Make sure that all information entered here is included in the Methods section of the manuscript.**	NA

Powered by Editorial Manager® and ProduXion Manager® from Aries Systems Corporation

**Format for specific study types**<br><br>**Human Subject Research (involving human participants and/or tissue)**<br>• Give the name of the institutional review board or ethics committee that approved the study<br>• Include the approval number and/or a statement indicating approval of this research<br>• Indicate the form of consent obtained (written/oral) or the reason that consent was not obtained (e.g. the data were analyzed anonymously)<br><br>**Animal Research (involving vertebrate animals, embryos or tissues)**<br>• Provide the name of the Institutional Animal Care and Use Committee (IACUC) or other relevant ethics board that reviewed the study protocol, and indicate whether they approved this research or granted a formal waiver of ethical approval<br>• Include an approval number if one was obtained<br>• If the study involved *non-human primates*, add *additional details* about animal welfare and steps taken to ameliorate suffering<br>• If anesthesia, euthanasia, or any kind of animal sacrifice is part of the study, include briefly which substances and/or methods were applied<br><br>**Field Research**<br><br>Include the following details if this study involves the collection of plant, animal, or other materials from a natural setting:<br>• Field permit number<br>• Name of the institution or relevant body that granted permission	
**Data Availability**<br><br>Authors are required to make all data underlying the findings described fully available, without restriction, and from the time of publication. PLOS allows rare exceptions to address legal and ethical concerns. See the PLOS Data Policy and FAQ for detailed information.	Yes - all data are fully available without restriction

A Data Availability Statement describing where the data can be found is required at submission. Your answers to this question constitute the Data Availability Statement and **will be published in the article**, if accepted.<br><br>**Important:** Stating 'data available on request from the author' is not sufficient. If your data are only available upon request, select 'No' for the first question and explain your exceptional situation in the text box.<br><br>Do the authors confirm that all data underlying the findings described in their manuscript are fully available without restriction?	
**Describe where the data may be found in full sentences. If you are copying our sample text, replace any instances of XXX with the appropriate details.**<br><br>• If the data are **held or will be held in a public repository**, include URLs, accession numbers or DOIs. If this information will only be available after acceptance, indicate this by ticking the box below. For example: *All XXX files are available from the XXX database (accession number(s) XXX, XXX.*).<br>• If the data are all contained **within the manuscript and/or Supporting Information files**, enter the following: *All relevant data are within the manuscript and its Supporting Information files.*<br>• If neither of these applies but you are able to provide **details of access elsewhere**, with or without limitations, please do so. For example:<br><br>*Data cannot be shared publicly because of [XXX]. Data are available from the XXX Institutional Data Access / Ethics Committee (contact via XXX) for researchers who meet the criteria for access to confidential data.*<br><br>*The data underlying the results presented in the study are available from (include the name of the third party*	All data have been added to manuscript as well as supplementary file.

and contact information or URL).<br>• This text is appropriate if the data are owned by a third party and authors do not have permission to share the data.<br><br>* typeset	
Additional data availability information:	

Manuscript Click here to access/download;Manuscript;manuscript.docx

Distribution and risk assessment of pesticide residues in sediment samples from river Ganga, India

Zeshan Umar Shah*, Saltanat Parveen*

***Limnology Research Laboratory, Department of Zoology, Aligarh Muslim University, Aligarh, 202002**

Corresponding authors email: zeshanomer11@gmail.com

Abstract:

Indiscriminate use of pesticides leads to their entry in to the bottom sediments, where they are absorbed in the sediment's particle and thus, may become the consistent source of aquatic pollution. The present work was carried out to evaluate pesticide residues in the sediment samples and associated human health risk of commonly used pesticides along the basin of river Ganga. Total of 16 pesticides were analyzed along three stretches of river Ganga. The concentration of pesticides in the upper stretch ranged from ND to 0.103 µg/kg, in the middle stretch ND to 0.112 µg/kg, and in the lower stretch ND to 0.105 µg/kg. Strong positive correlation was found between total organic carbon and total pesticide residues in sediment samples. Carcinogenic and non-carcinogenic values were estimated below the threshold limit suggesting no associated risk. Risks associated with the inhalation route of exposure were found to be higher than the dermal and ingestion routes. Children were found at higher risk at each site from multiple routes of exposure than adult population groups. Toxic unit values were found to be below the threshold value suggesting no risk associated with exposure of pesticides from sediments. However, long term effects on ecological quality due to consistent pesticide exposure must not be ignored. Therefore, the present study focuses on concrete efforts to reduce the threat of pesticide pollution in the river system and possible human health risk.

Keywords: River Ganga, Pesticides, Sediments, Ecological risk, Human health risk

Introduction:

Pesticides belong to the class of chemicals substances used to improve and increase crop production. However after use their residues tend to persist and accumulate in the different environmental compartments. In the aquatic ecosystem the rate of bio-accumulation depends on the solubility and octanol-water partition coefficient (logKow) of the pesticides. The residues present in the environment have impact on both environment and human health even at low concentration (Ccanccapa et al., 2016, Shah and Parveen 2021a, Shah and Parveen 2021b, Singh *et al*., 2012, Kuranchie et al., 2012). Furthermore, physical, chemical and biological agents of the environment degrade the parent compound into few or more components that become more persistent and toxic to organisms (Kumar and Sachan 2021, Ozkara *et al*., 2016, Conde-Avila *et al*., 2020, Kolankaya, 2006).

The potential ecotoxicological risks associated with pesticides are addressed with determination of Toxic units (TUs) and risk quotients (RQs) (EC, 2003, Ccanccapa *et al*., 2016, Ozekeke *et al*., 2019). Large numbers of studies considering the above criteria have been carried out in many countries (Teklu *et al*., 2015, Palma *et al*., 2014, Ozkara *et al*., 2016, Lehmann *et al*., 2017) including India (Mondal, 2018, Arisekar *et al*., 2021, Singare 2016, Sah *et al*., 2020, Shah and Parveen 2021). The pesticide residues also tend to accumulate in the sediments of the aquatic ecosystem by their absorption ~~and absorption in the sediment's particle~~ and thus, may become ~~the~~ consistent source of aquatic pollution. Therefore based on the guidelines of EC 2000, EC 2008, EU 2013, it is undoubtedly needed to include pesticide concentration in sediment for risk assessment. Although a variety of discussions and methods are available on sediment risk

assessment associated with pesticide toxicity, these are rarely applied (Lawrence *et al*., 2015, Ndunda *et al*., 2018, Ogbeide *et al*., 2016, Mondal *et al*., 2018).

The other problem usually discussed is the impact by mixture of different pesticides present at a time. Thus, ~~the~~ need to establish the real impact of these mixtures (pesticides) on biota communities of the ecosystem (Rodney *et al*., 2013, Lydy *et al*., 2004, Bighiu *et al*., 2020), which can be predicted by the independent action of the pesticide or by concentration addition impact. Consumers assume the different action of pesticides, therefore ignoring the agonistic/antagonistic action of mixture and overestimating the effect (Van Metre *et al*., 2017, Wilkinson *et al*., 2000, Altenburger *et al*., 1993). Concentration addition is often the recommended first step towards impact process as it provides the worst case scenario of mixture effect (de Castro-Català *et al*., 2016).

River Ganga is the largest and most affected river of India as large amounts of domestic and agricultural wastes runoff into the river. This raises a serious concern on concentrations of the cocktail of pesticides present in water (Shah and Parveen 2021). The rising concentration of pesticide residues in aquatic ecosystems leads to degradation and significant biodiversity loss (Ginebreda *et al*., 2014). In addition, human exposure to contaminated water via bath/swimming and ingestion poses serious risk (Chowdhury 2015, Lu et al., 2017). Therefore there is an increase in assessing and predicting the environmental risk of pesticides by employing ecotoxicological indices (Ccanccapa *et al*., 2016, Sangchan *et al*., 2014, Carazo-Rojas *et al*., 2018, Ogbeide *et al*., 2015, Mahmood *et al*., 2014, Ogbeide *et al*., 2018, Yun *et al*., 2014). Risk assessment involves estimation of the possible impact posed on non-target organisms upon environmental pesticide concentration exposure. The toxic unit (TUs) estimation is the deterministic approach towards the ecological risk assessment of pesticides to non-target aquatic

organisms. Toxic unit for sediment samples is determined by using the individual concentration of pesticide and its critical toxicological endpoint for the given organism (Sprague 1971).

Risk assessment involves assessment of potential risk posed to humans upon exposure to environmental contaminants. Estimating the risk of pesticides depends on the route of exposure to human body as contaminants may occur in different media sources of the aquatic ecosystem (Kim *et al*., 2017, Damalas and Koutroubas 2016, Mostafalou and Abdollahi 2017, Wang *et al*., 2013, McKinlay *et al*., 2008, Alshemmari *et al*., 2021, Yu *et al*., 2012). The exposure pathway to pesticides includes both dietary and non-dietary exposures (Teysseire *et al*., 2020, Jensen *et al*., 2015, Hubal *et al*., 2000). Dietary exposure route involves exposure to contaminants via food and water contaminated with pesticide residues while non-dietary exposure involves dermal contact with contaminated water and sediments while bathing/ swimming, inhalation of residues from sediments and probable ingestion of contaminated sediments (Ge *et al*., 2013, Sarwar 2015, Alshemmari *et al*., 2021).

The objective of this study was to establish the concentration of pesticide residues in sediment samples along three different stretches of river Ganga and based on these concentrations ecological risk on non-target organisms using deterministic approach Toxic Units (TUs) and determination of carcinogenic and non-carcinogenic risk via different exposure routes into the human body. Thus, the study is important as it mainly focuses on the deterministic and probabilistic approach towards risk assessment of pesticides pollution in sediments. Furthermore, the river plays an important socio-economic role in the development of the country as it is an essential habitat for diverse fishes and provides other ecosystem services.

Materials and Methods:

Sampling site and collection of sediments:

Three stations were selected along the whole length of river Ganga for pesticide analysis in the sediment samples (Figure 1). In the upper stretch Rishikesh station (30°03′51″N, 78°17′28″E), in the middle stretch Narora station (28°12′02″N, 78°23′41″E) and in the lower stretch Patna station (25°36′28″N, 85° 08′34″E). The river Ganga originates from Gangotri in the Himalayas and flows through highly populated agricultural areas of Uttarakhand, Uttar Pradesh and Bihar, the deposits of organic pollutants are drifted through surface runoff into the river water. The most cultivable vegetation includes paddy, wheat, sugar cane etc.

A total of 40 random samples from bottom (3-4 meters) sediments were collected using Ekman sediment grabber operated manually from the bridge and placed in aluminium foil (Yang *et al*., 2015). For further analysis, an ice box was used to store immediately and transport all samples to the laboratory.

Chemicals and Reagents:

Organic solvent n-hexane and acetone (HPLC grade) ~~was~~ purchased from Sigma Aldrich. Co., USA and ~~was~~ glass distilled before use. Anhydrous Sodium Sulphate (Na_2So_4) and Sulphuric acid (H_2So_4) ~~was~~ procured from Himedia Pvt. Ltd. New Delhi, India. Pesticides of purity (>99%) ~~was~~ purchased from Sigma Aldrich, USA and Rankem Pvt. Ltd. New Delhi, India.

Extraction of samples:

Pesticide residues in the sediment samples were extracted by Soxhlet extraction method (Mutua *et al*., 2015). 100 g of sediment of each sample was packed in blotting paper and transferred into a thimble fitted with a condenser and connected with a round bottom flask containing 150 ml of (1:1) hexane and acetone solvent. Solvent flask was refluxed for 8 hours at 85 °C. The extract

was finally concentrated on a water bath to 1 ml and redissolved in 100ml of distilled water and cleaned up by solid phase extraction method based on physical and chemical properties of compounds that needs to be separated.

Analysis of samples:

The equipment used in the analysis of pesticides in sediment samples was liquid chromatography equipped with a mass spectrometer (TripleTof 5600+Make Sciex). Cleaned extracts were used for analysis of pesticide residues. DB-5 (30m 0.25mm 0.25m film thickness) was used as the analytical capillary column. A flow rate of the eluent was maintained at 0.2 ml/min for 10 minutes. Analyte detection was carried out in the electron capture detection (ECD) mode. Presence of the quantification, confirmation ions and retention time was based on the authentic standards (Sigma Aldrich).

Quality control and quality assurance:

Analytes used were subjected to rigorous quality control and quality assurance methods for reliability of the results. HPLC grade reagents and double distilled deionized water was used throughout the experiment. Blank was always run for the correction of reading of the instrument. Internal standard ensures the accuracy of the extract and cleaning method. Limit of detection (LOD), limit of quantification (LOQ) and recovery rate was within the range of 75 to 90% (Table S2), peaks were identified by comparing internal standards with extract retention time.

Statistical analysis:

The results of the pesticide analysis obtained were presented as mean and standard deviation using Microsoft excel 2010 and visualized by Box and whisker plot. Significant differences in

concentration of pesticide residues were evaluated by using one way ANOVA (Microsoft excel 2010) and Duncan's Multiple Range Test (DMRT) (SPSS 16.0).

PCA analysis:

Pesticides (organochlorine, organophosphate, carbamate) detected in sediment samples were subjected to Principal Component Analysis (PCA) using SPSS 16 to infer hypothetical sources of pesticides. PCA was performed with varimax rotation because it minimizes the number of variables with high loading on each component, thus facilitating the interpretation of PCA results (Frimpong *et al*., 2013).

Health risk estimation:

In estimating the human health risk of pesticide residues observed in sediment samples of the three stretches of river Ganga, three possible routes of exposure were considered. The routes are: ingestion of contaminated sediments, dermal contact with contaminated sediments, and inhalation of residues from contaminated sediments. Specific risk assessment models have been used for this purpose.

Toxic Units:

The toxic units were used for the estimation of risk of pesticides in sediment samples for algae, daphnia and fish based on the method of (Ccanccapa *et al*., 2016). As per regulatory framework for chemicals (REACH) the toxicity tests for chemicals require different trophic organisms (primary producers, primary and secondary consumers) to protect more sensitive aquatic community

For the estimation of the toxic unit associated with sediment samples pore water concentration was estimated in the equilibrium-partitioning approach. This is the important approach towards establishing sediment toxicity benchmarks (Di Toro *et al.*, 1991). Pore water concentration (C_{pw}) in the sediment samples was estimated following the formula given below.

$$Cpw = \frac{Cs}{Kd}$$

Where Kd is partitioning coefficient, Cs is the sediment concentration, Cpw is pore water concentration of the pesticides.

Kd was estimated by the given formula

$$Kd = Koc \times foc$$

Where, Koc is the organic carbon water partitioning coefficient for the pesticides.

Foc is a fraction of the total organic carbon measured in the sediment samples.

Log Koc is the octanol-water partitioning coefficient and is determined by the formula given below.

$$\text{Log Koc} = a \times \text{Log Kow} + b$$

The constant (a) and (b) were set to be 0.72 and 0.49 Schwarzenbach and Westall 1981.

Chronic daily intake (CDI):

The United States Environmental Protection Agency (USEPA, 1997) has developed a model to estimate the non-carcinogenic risks of pesticide exposure to contaminants in adults and children

through dietary and non-dietary exposure. Human exposure to the pesticides can occur either through ingestion, through bathing or recreational activities.

River Ganga is the place where large numbers of devotees all around India are attracted for religious activities who come to take bath or swim. In addition a large percentage of water is used for irrigation and household usage. The formula given below were used to estimate non-carcinogenic risks.

Hazard quotient (HQ) = $\frac{\mathrm{CDI}}{\mathrm{RfD}}$

The carcinogenic risk for each pesticide was estimated using the cancer slope factor. The following formula was used.

R = CDI × SF

Where CDI is the chronic daily intake, whereas RfD and SF are individual pesticides reference dose and slope factor.

All the routes of exposure (dermal contact, ingestion and inhalation) were subjected to estimation of non-carcinogenic risk in adults and children. Exposure due to inhalation may occur when particular matter (dust), vapors or aerosols containing contaminant residues are released during low tides from dried sediments, exposure of dermal contact may occur by taking bath or swimming in the river, ingestion of pesticides may occur by drinking the water of eating food from the river (USEPA, 2017). For each population group formula 3-5 used to estimate the CDI of contaminated sediment samples was taken from Huang *et al*. (2014).

CDI ingestion = $\frac{\mathrm{C\ (sediment)} \times \mathrm{IR\ (sediment)} \times \mathrm{CF} \times \mathrm{EF} \times \mathrm{ED}}{\mathrm{BW} \times \mathrm{AT}}$ …...3

$$\text{CDI inhalation} = \frac{\text{C (sediment)} \times \left(\frac{1}{\text{PET}}\right) \times \text{IAR} \times \text{EF} \times \text{ED}}{\text{BW} \times \text{AT}} \quad \ldots\ldots.4$$

$$\text{CDI dermal} = \frac{\text{C (sediment)} \times \text{SA} \times \text{CF} \times \text{EF} \times \text{ED} \times \text{ABS} \times \text{AF}}{\text{BW} \times \text{AT}} \quad \ldots\ldots5$$

Results and discussion:

Contamination of sediment with pesticides is a common feature of the riverine system that drains large areas of intense agricultural fields. Sediment analysis of river Ganga showed a diverse classes of pesticides at varying concentrations (Table 1). The sediment samples collected from three different stations were analyzed for 15 pesticides. In the upper stretch (Figure 2) highest mean concentration was methylparathion (0.103 µg/kg dry weight (dw)), while the lowest mean concentration being nuarimol (0.056 µg/kg dw). Chlordane, heptachlor, methoxychlor, dichlorvos and dimethoate were not found in the sediment samples. The concentration of other pesticides was malathion (0.096 µg/kg dw), azinphosmethyl (0.084 µg/kg dw), tridemorph (0.070 µg/kg dw) and cypermethrin (0.063 µg/kg dw). Maximum number of pesticides was found at Narora station (Figure 3) of middle stretch with highest mean concentration being cypermethrin (0.112 µg/kg dw), while the lowest mean concentration being nuarimol (0.062 µg/kg dw). The most ubiquitous pesticides were tridemorph (0.107 µg/kg dw), dichlorvos (0.099 µg/kg dw), chlordane (0.098 µg/kg dw), atrazine (0.096 µg/kg dw) and malathion (0.093 µg/kg dw). In the lower stretch (Figure 4) highest mean concentration being chlordane (0.105 µg/kg dw), while the lowest mean concentration being methylparathion (0.073 µg/kg dw). Atrazine was not detected in the analyzed samples. The concentration of other pesticides was methoxychlor 0.103 µg/kg dw, dichlorvos 0.102 µg/kg dw, cypermethrin 0.096 µg/kg dw, azinphosmethyl 0.093 µg/kg dw, tridemorph 0.092 µg/kg dw, binapacryl 0.091 µg/kg dw and nuarimol 0.084

μg/kg dw. The detected pesticides have high octanol/water partition coefficient (log k_{ow}), hydrophobic, low water solubility and therefore tend to accumulate more in the sediments. Other factors including time and rate of consumption are also important in the accumulation of pesticides. The findings in our study are in partial accordance with the finding of Singh *et al*., 2012 who have observed lindane, methylparathion, endosulfan and DDT from Champanala, Mond ghat and Burning ghat at Bhagalpur station of river Ganga. Malik *et al*., 2009 have observed similar types of pesticides heptachlor (3.85 ng/g) and methoxychlor (0.47 ng/g) from sediment samples of Gomti, a tributary of river Ganga. Mondal *et al*., 2018 have observed a class of persistent organochlorine and organophosphate pesticides from sediment analysis of Hooghly River in West Bengal.

Majority of chemical pollutants discharged into aquatic resources end up concentrated in sediments that act as sink as well as source of the toxicity. The results of this study highly contribute to the fact that sediments play an important role in retention of pesticides in the aquatic ecosystem. Large proportion of available pesticides bind to suspended particles and settle down at the bottom of the river (Li *et al*., 2014, Olutona *et al*., 2014). Presence of pesticide residues in the sediments can inhibit microbial processes thus affecting the degradation rate of organic matter, which could result in generation of anaerobic gasses that affect growth of ecosystem (Staley *et al*., 2015). There was a positive correlation between total organic carbon and pesticide concentration in sediments. Which indicates that sediment organic carbon could enhance adsorption and deposition of pesticide residues because of their hydrophobicity (Figure 5). The total organic carbon percentage in the sediments collected from three stations vary, in the upper stretch mean percentage was 1.67%. In the middle and lower stations mean percentage were 1.88 and 1.89%. Therefore higher amounts of TOC were mentioned in middle and lower

stretches, thus pesticide deposition was higher than upper stretch. The increase of organic matter content in soil can supply more carbon source to facilitate microbial degradation of pesticide residues (Ding and Wu. 1997). As a result, the content of TOC could have an impact on the residues of pesticides in soil (Gong *et al*. 2004; Kalbitz *et al*. 1997). These results are similar to the findings of Devi *et al*. (2013) who reported significant correlation between pesticide and total organic carbon from surface soil collected from three major states of north eastern parts of India. Ogbeide *et al*., 2015 also reported positive correlation between total organic carbon and pesticide concentration in sediments collected from Owan River, Edo State Nigeria. It can be suggested that the high contribution of pesticides in sediments has probably originated from a similar contribution source. It has been already reported that the higher the value of organic carbon higher the partition coefficients (K_{OC}), suggesting that these compounds get strongly adsorbed in the sediments (Doucette, 2003).

Pesticide residue levels found in this study were compared with various studies from the world (Table S1). The observed concentrations in this study were lower than the several studies from China (Yang *et al*., 2005, Zhou *et al*., 2006, Sun *et al*., 2010) and Niger river Nigeria (Unyimadu et al., 2019). While these fall in the range of those observed from Ikpoda River and Ebro River from Nigeria (Ogbeide *et al*., 2019) and Spain (Ccanccapa *et al*., 2016). The concentrations were above the levels observed from the urban river in Florida and four urban creeks in California, USA (Yang *et al*., 2015, Phillips *et al*., 2010) and Lake Tashk, Iran (Kafilzadeh *et al*., 2015) (Table S1).

Principal component analysis of sediment samples is present in (Figure 6). The figure shows two components that account for 75.90% of the total variance. PC1 (56.7 %) is explained by positive correlation loading for the variables: heptachlor, dichlorvos, dimethoate, nuarimol, tridemorph,

methoxychlor and cypermethrin. PC2 (43.2%) is positively loaded for malathion and atrazine. The positive loading of malathion in PC2 indicates that it has different degradation and distribution pattern. Positive loading of pesticide residues in a component implies that these residues undergo a similar degradation and distribution pattern in the medium (Kilulya and Mhinzi, 2012, Lawrence *et al*., 2015, Ogbeide *et al*., 2015).

The determination of carcinogenic and non-carcinogenic risk associated with pesticide exposure from sediments includes three routes (Table S3 and S4, USEPA). The non-carcinogenic risk estimation from the three stations (Table 2,3,4) were below the reference levels (USEPA) for each studied pesticide, therefore these contaminants are unlikely considered to pose potential risk to human health (Huang *et al*., 2014). According to the USEPA, guidelines if the estimated CDI value of pesticide exceeds oral reference dose (Table S3, USEPA) value is supposed to pose adverse health impact or non-carcinogenic risk to human health. The estimated CDI value for each pesticide showed that children are at higher risk than adults. Ogbeide *et al*., 2016 have reported similar results for CDI values from Illushi River, Ogbesse River and Owan River of Nigeria. Carcinogenic risk from the exposure of pesticides in sediments was below the threshold limit of 10^{-6} (USEPA) indicating no observed risk for both adults and children population groups at each station. Estimated results for non-carcinogenic and carcinogenic risk showed, pesticide exposure from the inhalation route has higher potential to cause risk in adults and children than by other two routes. Further, the study also clarifies that there is a higher potential of cancer risk through multiple exposure routes in children than in adult population groups.

The toxic unit (TU) is used to estimate the potential ecological risk of pesticide residue in sediments (Table 5). The observed toxic units for sediments were <1 which indicates that there is no potential acute risk with presence of pesticide residues in sediments. The observed results in

this study are in accordance with Ogbeide *et al*. (2019) and Ccanccapa *et al*. (2016) who reported sediment toxic units <1 for pesticides in Ikpoda river from Nigeria and Ebro River from Spain. ~~Although~~ the observed results are below the threshold value, which suggest no acute effect due to the presence of pesticides. However complex chronic effects and potential loss of ecological quality cannot be ignored. The pattern of pesticide pollution, including its impact on species, could be attributed to the recent decline in abundance of species diversity as reported by Sarkar *et al*. (2010).

Conclusion:

The study carried out aimed to provide information regarding the distribution and risk of commonly used pesticides along the basin of river Ganga. Observed concentrations of studied pesticides along the three stations may have potential of chronic effect to both the aquatic species and associated humans. Standard protocol developed for carcinogenic and non-carcinogenic risk assessment is considered as tool to give the probable risk associated with pesticide exposure from sediments. The estimated results showed no acute risk of cancer and non-cancer effects from multiple routes (ingestion, dermal contact and inhalation) of exposure to pesticides present in sediments to both adult and children groups of populations. Probabilistic results showed pesticide exposure from inhalation would have higher risk to both population groups, nevertheless children are at higher risk through multiple routes of exposure than adults. Probable ecological risk assessment was determined by toxic unit values and was found to have a potential risk to the aquatic organisms inhabiting the ecosystem. Results from this study highlight the need of concrete steps to be taken for regular monitoring and pesticide pollution control along the river basin, thus, to reduce the associated risk on aquatic ecosystems and associated humans.

Acknowledgement:

Authors would like to acknowledge Chairperson Department of Zoology, Aligarh Muslim University, Aligarh for providing requisite facilities; simultaneously authors like to acknowledge USIF AMU, Aligarh for providing LCMS faculties to carry of this work.

References:

Alshemmari, H., Al-Shareedah, A.E., Rajagopalan, S., Talebi, L.A. and Hajeyah, M., 2021. Pesticides driven pollution in Kuwait: The first evidence of environmental exposure to pesticides in soils and human health risk assessment. *Chemosphere*, *273*, p.129688.

Altenburger, R., Boedeker, W., Faust, M. and Grimme, L.H., 1993. Aquatic toxicology, analysis of combination effects. *Handbook of hazardous materials*, pp.15-27.

Arisekar, U., Shakila, R.J., Shalini, R. and Jeyasekaran, G., 2021. Pesticides contamination in the Thamirabarani, a perennial river in peninsular India: The first report on ecotoxicological and human health risk assessment. *Chemosphere*, *267*, p.129251.

Bighiu, M.A., Gottschalk, S., Arrhenius, Å. and Goedkoop, W., 2020. Pesticide Mixtures Cause Short- Term, Reversible Effects on the Function of Autotrophic Periphyton Assemblages. *Environmental toxicology and chemistry*, *39*(7), pp.1367-1374.

Carazo-Rojas, E., Pérez-Rojas, G., Pérez-Villanueva, M., Chinchilla-Soto, C., Chin-Pampillo, J.S., Aguilar-Mora, P., Alpízar-Marín, M., Masís-Mora, M., Rodríguez-Rodríguez, C.E. and Vryzas, Z., 2018. Pesticide monitoring and ecotoxicological risk assessment in surface water bodies and sediments of a tropical agro-ecosystem. *Environmental pollution*, *241*, pp.800-809.

Ccanccapa, A., Masiá, A., Navarro-Ortega, A., Picó, Y. and Barceló, D., 2016. Pesticides in the Ebro River basin: occurrence and risk assessment. *Environmental Pollution*, *211*, pp.414-424.

Chowdhury, S., 2015. Predicting human exposure and risk from chlorinated indoor swimming pool: a case study. *Environmental monitoring and assessment*, *187*(8), pp.1-14.

Conde-Avila, V., Ortega-Martínez, L.D., Loera, O., El Kassis, E.G., Dávila, J.G., Valenzuela, C.M. and Armendáriz, B.P., 2020. Pesticides degradation by immobilised microorganisms. *International Journal of Environmental Analytical Chemistry*, pp.1-31.

Damalas, C.A. and Koutroubas, S.D., 2016. Farmers' exposure to pesticides: toxicity types and ways of prevention.

de Castro-Català, N., Kuzmanovic, M., Roig, N., Sierra, J., Ginebreda, A., Barceló, D., Pérez, S., Petrovic, M., Picó, Y., Schuhmacher, M. and Muñoz, I., 2016. Ecotoxicity of sediments in rivers: Invertebrate community, toxicity bioassays and the toxic unit approach as complementary assessment tools. *Science of the total environment*, *540*, pp.297-306.

Devi, N.L., Chakraborty, P., Shihua, Q. and Zhang, G., (2013). Selected organochlorine pesticides (OCPs) in surface soils from three major states from the northeastern part of India. *Environmental monitoring and assessment*, **185**(8): 6667-6676.

Ding, J.Y. and Wu, S.C., (1997). Transport of organochlorine pesticides in soil columns enhanced by dissolved organic carbon. *Water Science and technology*, **35**(7): 139-145.

Doucette, W.J. (2003). Quantitative structure- activity relationships for predicting soil- sediment sorption coefficients for organic chemicals. *Environmental Toxicology and Chemistry: An International Journal*, **22**(8): 1771-1788.

Frimpong, S.K., Yeboah, P.O. and Fletcher, J.J. (2013). Distribution pattern of pesticide residues in cocoa beans across the cocoa growing regions of Ghana.

Ge, J., Woodward, L.A., Li, Q.X. and Wang, J., 2013. Composition, distribution and risk assessment of organochlorine pesticides in soils from the Midway Atoll, North Pacific Ocean. *Science of the total environment*, *452*, pp.421-426.

Ginebreda, A., Kuzmanovic, M., Guasch, H., de Alda, M.L., López-Doval, J.C., Muñoz, I., Ricart, M., Romaní, A.M., Sabater, S. and Barceló, D., 2014. Assessment of multi-chemical pollution in aquatic ecosystems using toxic units: compound prioritization, mixture characterization and relationships with biological descriptors. *Science of the total environment*, *468*, pp.715-723.

Gong, Z.M., Tao, S., Xu, F.L., Dawson, R., Liu, W.X., Cui, Y.H., Cao, J., Wang, X.J., Shen, W.R., Zhang, W.J. and Qing, B.P. (2004). Level and distribution of DDT in surface soils from Tianjin, China. *Chemosphere*, **54**(8):1247-1253.

Huang, T., Guo, Q., Tian, H., Mao, X., Ding, Z., Zhang, G., Li, J., Ma, J. and Gao, H., 2014. Assessing spatial distribution, sources, and human health risk of organochlorine pesticide residues in the soils of arid and semiarid areas of northwest China. *Environmental Science and Pollution Research*, *21*(9), pp.6124-6135.

Hubal, E.A.C., Sheldon, L.S., Zufall, M.J., Burke, J.M. and Thomas, K.W., 2000. The challenge of assessing children's residential exposure to pesticides. *Journal of Exposure Science & Environmental Epidemiology*, *10*(6), pp.638-649.

Jensen, B.H., Petersen, A., Nielsen, E., Christensen, T., Poulsen, M.E. and Andersen, J.H., 2015. Cumulative dietary exposure of the population of Denmark to pesticides. *Food and Chemical Toxicology*, *83*, pp.300-307.

Kafilzadeh, F., 2015. Assessment of organochlorine pesticide residues in water, sediments and fish from Lake Tashk, Iran. *Achievements in the Life Sciences*, *9*(2), pp.107-111.

Kafilzadeh, F., Shiva, A.H., Malekpour, R. and Azad, H.N., 2012. Determination of organochlorine pesticide residues in water, sediments and fish from Lake Parishan, Iran. *World Journal of Fish and Marine Sciences*, *4*(2), pp.150-154.

Kalbitz, K., Popp, P., Geyer, W. and Hanschmann, G. (1997). β-HCH mobilization in polluted wetland soils as influenced by dissolved organic matter. *Science of the Total Environment*, **204**(1): 37-48.

Kilulya, K.F. and Mhinzi, G.S. (2012). Evaluation of patterns and spatial trends of pesticide residues from Vikuge Farm, Coast Region, Tanzania By principal components analysis. *Tanzania Journal of Science*, **38**(3): 184-200.

Kim, K.H., Kabir, E. and Jahan, S.A., 2017. Exposure to pesticides and the associated human health effects. *Science of the total environment*, *575*, pp.525-535.

Kolankaya, D., 2006. Organochlorine pesticide reidues and their toxic effects on the environment and organisms in Turkey. *International Journal of Environmental Analytical Chemistry*, *86*(1-2), pp.147-160.

Kumar, P. and Sachan, S.G., 2021. Exploring microbes as bioremediation tools for the degradation of pesticides. In *Advanced Oxidation Processes for Effluent Treatment Plants* (pp. 51-67). Elsevier.

Kuranchie-Mensah, H., Atiemo, S.M., Palm, L.M.N.D., Blankson-Arthur, S., Tutu, A.O. and Fosu, P., 2012. Determination of organochlorine pesticide residue in sediment and water from the Densu river basin, Ghana. *Chemosphere*, *86*(3), pp.286-292.

Lawrence, E., Ozekeke, O. and Isioma, T., 2015. Distribution and ecological risk assessment of pesticide residues in surface water, sediment and fish from Ogbesse River, Edo State, Nigeria. *Journal of Environmental Chemistry and Ecotoxicology*, *7*(2), pp.20-30.

Lehmann, E., Turrero, N., Kolia, M., Konaté, Y. and De Alencastro, L.F., 2017. Dietary risk assessment of pesticides from vegetables and drinking water in gardening areas in Burkina Faso. *Science of the Total Environment*, *601*, pp.1208-1216.

Li, Y., Niu, J., Shen, Z., Zhang, C., Wang, Z. and He, T., 2014. Spatial and seasonal distribution of organochlorine pesticides in the sediments of the Yangtze Estuary. *Chemosphere*, *114*, pp.233-240.

Lu, J., Mao, H., Li, H., Wang, Q. and Yang, Z., 2017. Occurrence of and human exposure to parabens, benzophenones, benzotriazoles, triclosan and triclocarban in outdoor swimming pool water in Changsha, China. *Science of the Total Environment*, *605*, pp.1064-1069.

Lydy, M., Belden, J., Wheelock, C., Hammock, B. and Denton, D., 2004. Challenges in regulating pesticide mixtures. *Ecology and Society*, *9*(6).

Mahmood, A., Malik, R.N., Li, J. and Zhang, G., 2014. Levels, distribution pattern and ecological risk assessment of organochlorines pesticides (OCPs) in water and sediments from two tributaries of the Chenab River, Pakistan. *Ecotoxicology*, *23*(9), pp.1713-1721.

Malik, A., Ojha, P. and Singh, K.P., 2009. Levels and distribution of persistent organochlorine pesticide residues in water and sediments of Gomti River (India)—a tributary of the Ganges River. *Environmental Monitoring and Assessment*, *148*(1), pp.421-435.

McKinlay, R., Plant, J.A., Bell, J.N.B. and Voulvoulis, N., 2008. Calculating human exposure to endocrine disrupting pesticides via agricultural and non-agricultural exposure routes. *Science of the Total Environment*, *398*(1-3), pp.1-12.

Mondal, R., Mukherjee, A., Biswas, S. and Kole, R.K., 2018. GC-MS/MS determination and ecological risk assessment of pesticides in aquatic system: A case study in Hooghly River basin in West Bengal, India. *Chemosphere*, *206*, pp.217-230.

Mondal, R., Mukherjee, A., Biswas, S. and Kole, R.K., 2018. GC-MS/MS determination and ecological risk assessment of pesticides in aquatic system: A case study in Hooghly River basin in West Bengal, India. *Chemosphere*, *206*, pp.217-230.

Mostafalou, S. and Abdollahi, M., 2017. Pesticides: an update of human exposure and toxicity. *Archives of toxicology*, *91*(2), pp.549-599.

Ndunda, E.N., Madadi, V.O. and Wandiga, S.O., 2018. Organochlorine pesticide residues in sediment and water from Nairobi River, Kenya: levels, distribution, and ecological risk assessment. *Environmental Science and Pollution Research*, *25*(34), pp.34510-34518.

Ogbeide, O., Chukwuka, A., Tongo, I. and Ezemonye, L., 2018. Relationship between geosorbent properties and field-based partition coefficients for pesticides in surface water and sediments of selected agrarian catchments: implications for risk assessment. *Journal of environmental management*, *217*, pp.23-37.

Ogbeide, O., Tongo, I. and Ezemonye, L., 2015. Risk assessment of agricultural pesticides in water, sediment, and fish from Owan River, Edo State, Nigeria. *Environmental monitoring and assessment*, *187*(10), pp.1-16.

Ogbeide, O., Tongo, I. and Ezemonye, L., 2016. Assessing the distribution and human health risk of organochlorine pesticide residues in sediments from selected rivers. *Chemosphere*, *144*, pp.1319-1326.

Ogbeide, O., Uhunamure, G., Okundaye, F. and Ejeomo, C., 2019. First report on probabilistic risk assessment of pesticide residues in a riverine ecosystem in South-South Nigeria. *Chemosphere*, *231*, pp.546-561.

Olutona, G.O., Ayano, S.A. and Obayomi-Davies, O., 2014. Organochlorine pesticide in water and bottom sediment from Aiba reservoir (Southwestern Nigeria). *Chemistry and Ecology, 30*(6), pp.513-531.

Özkara, A., Akyıl, D. and Konuk, M., 2016. Pesticides, environmental pollution, and health. In *Environmental health risk-hazardous factors to living species*. IntechOpen.

Palma, P., Köck-Schulmeyer, M., Alvarenga, P., Ledo, L., Barbosa, I.R., De Alda, M.L. and Barceló, D., 2014. Risk assessment of pesticides detected in surface water of the Alqueva

reservoir (Guadiana basin, southern of Portugal). *Science of the Total Environment*, *488*, pp.208-219.

Phillips, B.M., Anderson, B.S., Voorhees, J.P., Hunt, J.W., Holmes, R.W., Mekebri, A., Connor, V. and Tjeerdema, R.S., 2010. The contribution of pyrethroid pesticides to sediment toxicity in four urban creeks in California, USA. *Journal of Pesticide Science*, pp.1007230149-1007230149.

Rodney, S.I., Teed, R.S. and Moore, D.R., 2013. Estimating the toxicity of pesticide mixtures to aquatic organisms: a review. *Human and Ecological Risk Assessment: An International Journal*, *19*(6), pp.1557-1575.

Sah, R., Baroth, A. and Hussain, S.A., 2020. First account of spatio-temporal analysis, historical trends, source apportionment and ecological risk assessment of banned organochlorine pesticides along the Ganga River. *Environmental Pollution*, *263*, p.114229.

Sangchan, W., Bannwarth, M., Ingwersen, J., Hugenschmidt, C., Schwadorf, K., Thavornyutikarn, P., Pansombat, K. and Streck, T., 2014. Monitoring and risk assessment of pesticides in a tropical river of an agricultural watershed in northern Thailand. *Environmental monitoring and assessment*, *186*(2), pp.1083-1099.

Sarkar, U.K., Gupta, B.K. and Lakra, W.S., 2010. Biodiversity, ecohydrology, threat status and conservation priority of the freshwater fishes of river Gomti, a tributary of river Ganga (India). *The Environmentalist*, *30*(1), pp.3-17.

Sarwar, M., 2015. The dangers of pesticides associated with public health and preventing of the risks. *International Journal of Bioinformatics and Biomedical Engineering*, *1*(2), pp.130-136.

Shah, Z.U. and Parveen, S., 2021. Pesticide residues in Rita rita and Cyprinus carpio from river Ganga, India, and assessment of human health risk. *Toxicology Reports*, *8*, pp.1638-1644.

Shah, Z.U. and Parveen, S., 2021. Pesticides pollution and risk assessment of river Ganga: A review. *Heliyon*, *7*(8), p.e07726.

Singare, P.U., 2016. Distribution and risk assessment of suspected endocrine-disrupting pesticides in creek water of Mumbai, India. *Marine pollution bulletin*, *102*(1), pp.72-83.

Singh, L., Choudhary, S.K. and Singh, P.K., 2012. Pesticide concentration in water and sediment of River Ganga at selected sites in middle Ganga plain. *International journal of environmental sciences*, *3*(1), pp.260-274.

Sprague, J.B., 1971. Measurement of pollutant toxicity to fish—III: Sublethal effects and "safe" concentrations. *Water research*, *5*(6), pp.245-266.

Staley, Z.R., Harwood, V.J. and Rohr, J.R., 2015. A synthesis of the effects of pesticides on microbial persistence in aquatic ecosystems. *Critical reviews in toxicology*, *45*(10), pp.813-836.

Sun, J., Feng, J., Liu, Q. and Li, Q., 2010. Distribution and sources of organochlorine pesticides (OCPs) in sediments from upper reach of Huaihe River, East China. *Journal of Hazardous Materials*, *184*(1-3), pp.141-146.

Syed, J.H., Malik, R.N. and Muhammad, A., 2014. Organochlorine pesticides in surface soils and sediments from obsolete pesticides dumping site near Lahore city, Pakistan: contamination status and their distribution. *Chemistry and Ecology*, *30*(1), pp.87-96.

Teklu, B.M., Adriaanse, P.I., Ter Horst, M.M., Deneer, J.W. and Van den Brink, P.J., 2015. Surface water risk assessment of pesticides in Ethiopia. *Science of the Total Environment*, *508*, pp.566-574.

Teysseire, R., Manangama, G., Baldi, I., Carles, C., Brochard, P., Bedos, C. and Delva, F., 2020. Determinants of non-dietary exposure to agricultural pesticides in populations living close to fields: A systematic review. *Science of the Total Environment*, p.143294.

Tokatli, C., 2020. Pesticide Residues in Water and Sediment of Ergene River and Tributaries in Turkey. *Sigma*, *38*(1), pp.361-370.

Unyimadu, J.P., Osibanjo, O. and Babayemi, J.O., 2019. Concentration and distribution of organochlorine pesticides in sediments of the Niger River, Nigeria. *Journal of Health and Pollution*, *9*(22).

Van Metre, P.C., Alvarez, D.A., Mahler, B.J., Nowell, L., Sandstrom, M. and Moran, P., 2017. Complex mixtures of Pesticides in Midwest US streams indicated by POCIS time-integrating samplers. *Environmental Pollution*, *220*, pp.431-440.

Wang, W., Huang, M.J., Wu, F.Y., Kang, Y., Wang, H.S., Cheung, K.C. and Wong, M.H., 2013. Risk assessment of bioaccessible organochlorine pesticides exposure via indoor and outdoor dust. *Atmospheric environment*, *77*, pp.525-533.

Wilkinson, C.F., Christoph, G.R., Julien, E., Kelley, J.M., Kronenberg, J., McCarthy, J. and Reiss, R., 2000. Assessing the risks of exposures to multiple chemicals with a common mechanism of toxicity: how to cumulate?. *Regulatory Toxicology and Pharmacology*, *31*(1), pp.30-43.

Yang, R.Q., Lv, A.H., Shi, J.B. and Jiang, G.B., 2005. The levels and distribution of organochlorine pesticides (OCPs) in sediments from the Haihe River, China. *Chemosphere*, *61*(3), pp.347-354.

Yang, Y.Y., Toor, G.S. and Williams, C.F., 2015. Pharmaceuticals and organochlorine pesticides in sediments of an urban river in Florida, USA. *Journal of Soils and Sediments*, *15*(4), pp.993-1004.

Yu, Y., Li, C., Zhang, X., Zhang, X., Pang, Y., Zhang, S. and Fu, J., 2012. Route-specific daily uptake of organochlorine pesticides in food, dust, and air by Shanghai residents, China. *Environment international*, *50*, pp.31-37.

Yun, X., Yang, Y., Liu, M. and Wang, J., 2014. Distribution and ecological risk assessment of organochlorine pesticides in surface sediments from the East Lake, China. *Environmental Science and Pollution Research*, *21*(17), pp.10368-10376.

Zhou, R., Zhu, L., Yang, K. and Chen, Y., 2006. Distribution of organochlorine pesticides in surface water and sediments from Qiantang River, East China. *Journal of hazardous materials*, *137*(1), pp.68-75.

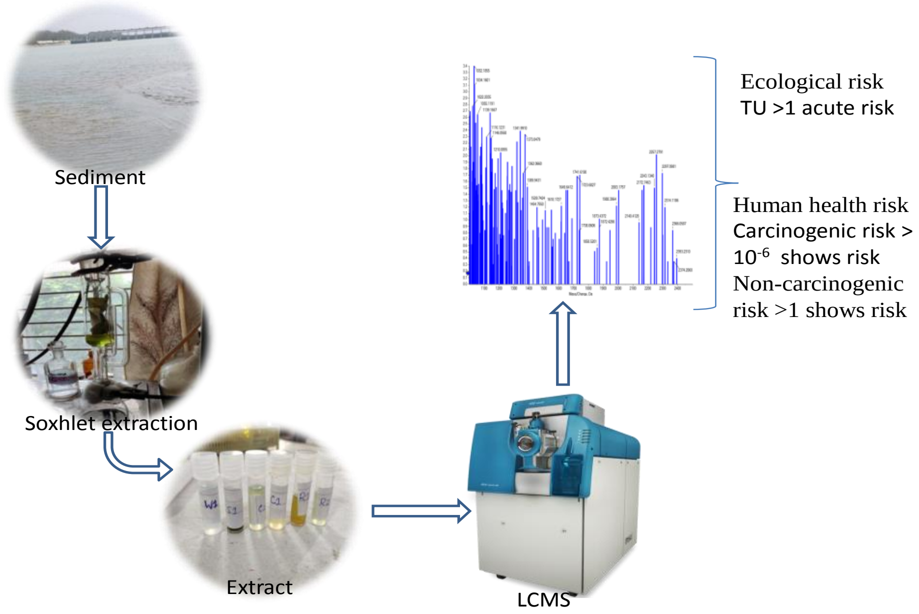

Graphical abstract

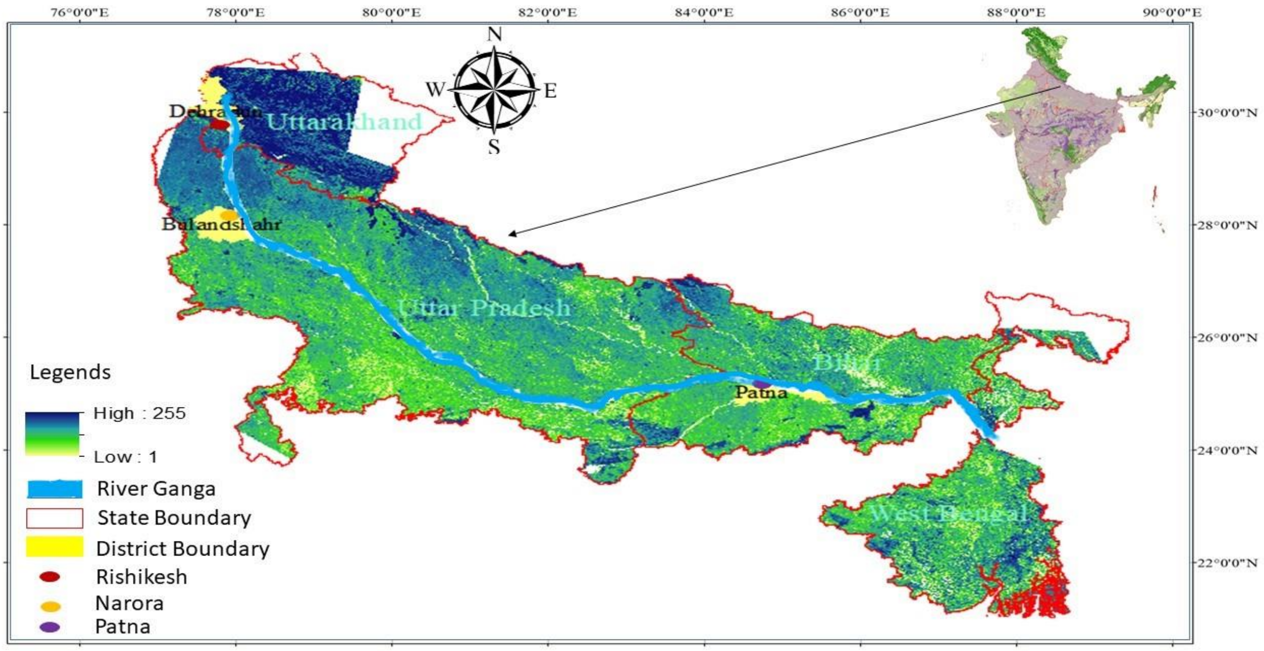

Figure 1: Map of study area showing the location of sampling stations along the river Ganga.

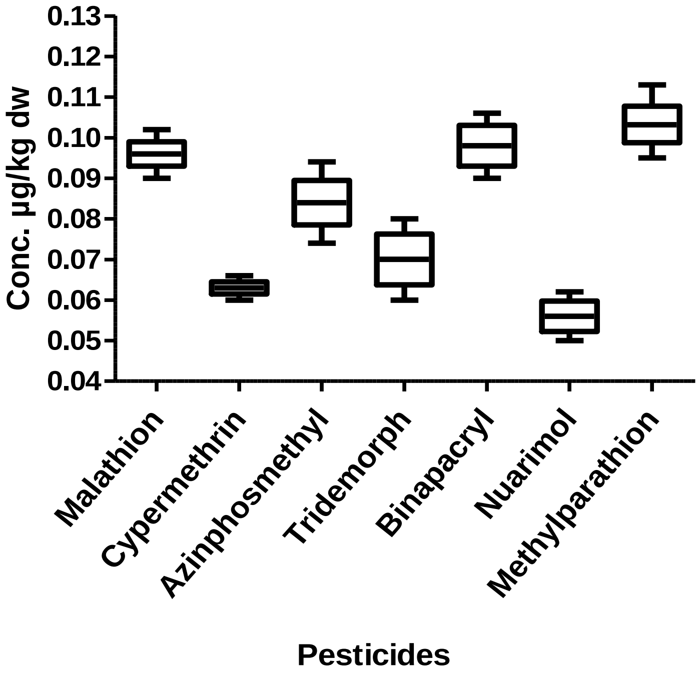

Figure 2: Box and whisker's plot of pesticide residues in sediment samples from upper stretch of river Ganga.

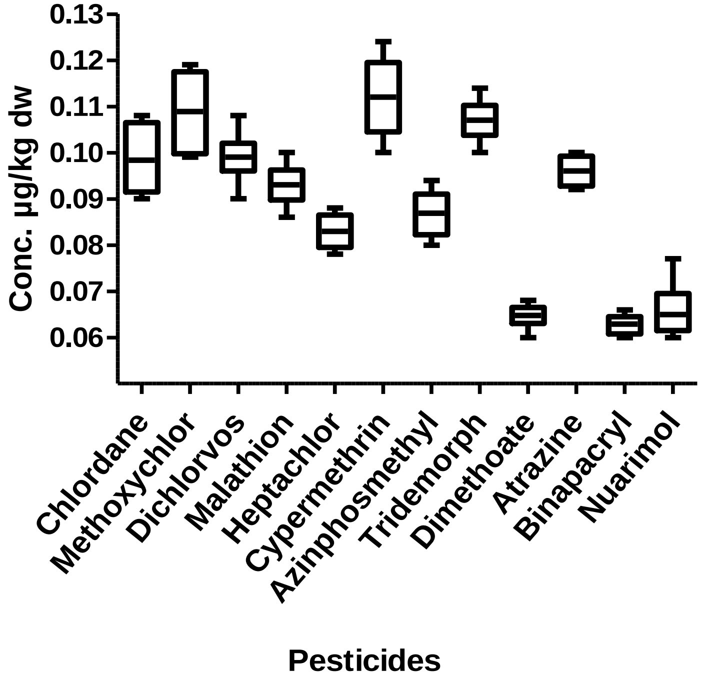

Figure 3: Box and whisker's plot of pesticide residues in sediment samples from middle stretch of river Ganga.

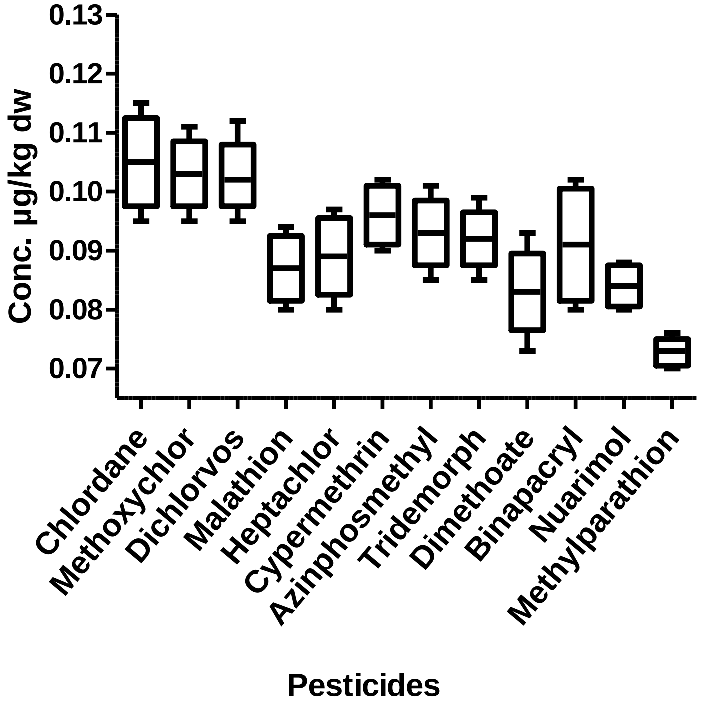

Figure 4: Box and whisker's plot of pesticide residues in sediment samples from lower stretch of river Ganga.

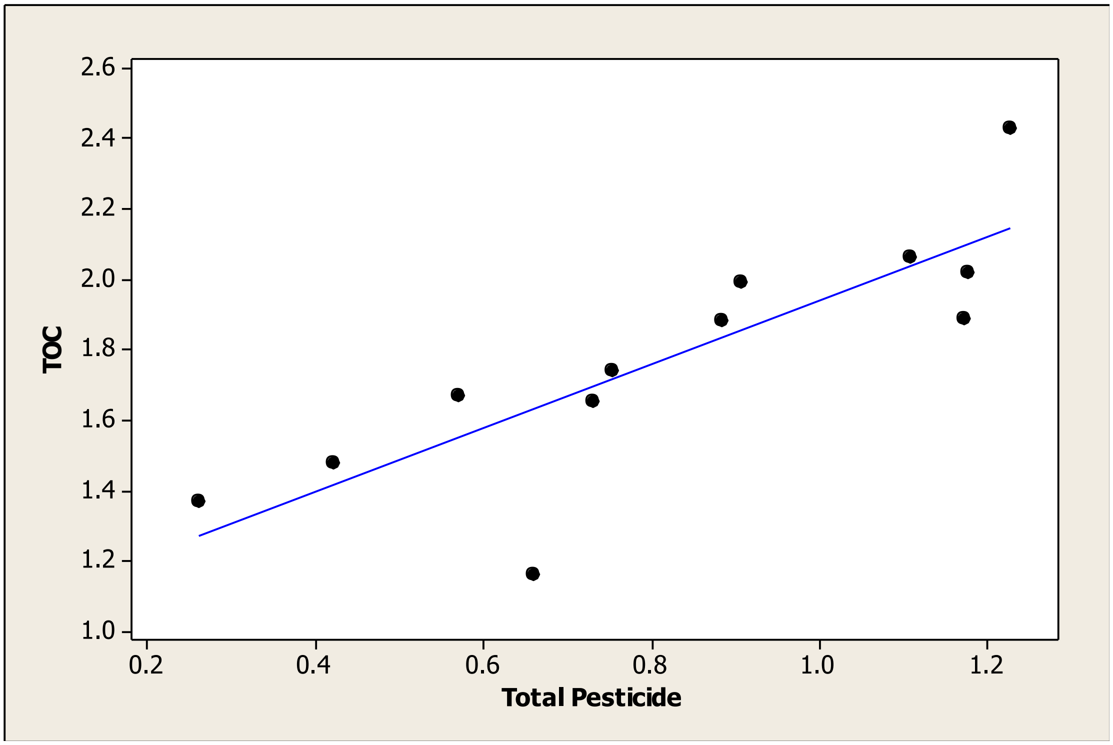

Figure 5: Correlation between total organic carbon and total pesticide concentration in sediment samples collected from three stations of river Ganga (r^2 = 0.8).

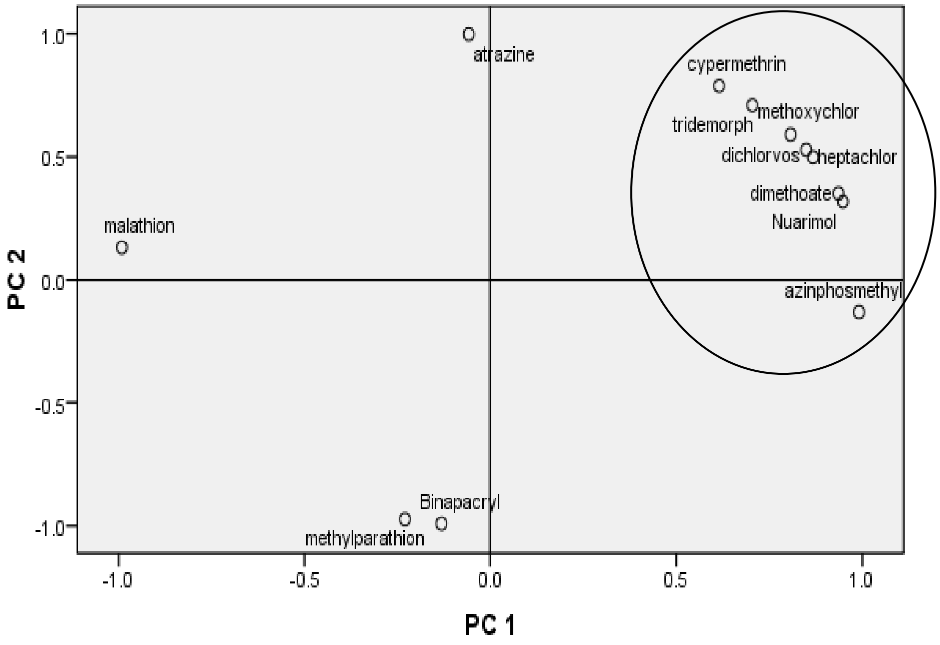

Figure 6: Principal component plot of pesticide residues in sediment samples from river Ganga.

Table 1: Pesticide residues detected in the sediment samples from three stations with (±) standard deviation, Mean with similar superscript in rows are insignificant at $P \leq 0.05$.

Pesticide	Middle stretch	Upper stretch	Lower stretch
Chlordane	0.098^{b} ± 0.007	nd	0.105^{a} ± 0.007
Methoxychlor	0.109^{a} ± 0.008	nd	0.103^{a} ± 0.005
Dichlorvos	0.099^{ab} ± 0.005	nd	0.102^{a} ± 0.005
Malathion	0.093^{a} ± 0.004	0.096^{a} ± 0.004	0.087^{b} ± 0.005
Heptachlor	0.083^{b} ± 0.003	Nd	0.089^{a} ± 0.006
Cypermethrin	0.112^{a}± 0.008	0.063^{c} ± 0.002	0.096^{b} ± 0.004
Azinphosmethyl	0.087^{b} ± 0.004	0.084^{b} ± 0.006	0.093^{a} ± 0.005
Tridemorph	0.107^{a} ± 0.004	0.070^{c} ± 0.007	0.092^{b} ± 0.004
Dimethoate	0.064^{b} ± 0.002	nd	0.083^{a} ± 0.006
Atrazine	0.096^{a} ± 0.003	nd	Nd
Chlormequat	Nd	nd	Nd
Fenobucarb	Nd	nd	Nd
Ethylthiourea	Nd	nd	Nd
Binapacryl	0.063^{c} ± 0.002	0.098^{a} ± 0005	0.091^{b} ± 0.008
Nuarimol	0.062^{b} ± 0.005	0.056^{c} ± 0.004	0.084^{a} ± 0.003
Methyl parathion	Nd	0.103^{a} ± 0.006	0.073^{b} ± 0.002

Table 2: Estimation of Chronic daily intake (CDI) and hazard quotient (HQ) for pesticide residues in sediment from middle stretch.

	Non-carcinogenic						Carcinogenic					
	Adult			Child			Adult			Child		
	Ingestion	Inhalation	Dermal	Ingestion	Inhalation	Dermal	Ingestion	Inhalation	Dermal	Ingestion	Inhalation	Dermal
Chlordane	2.23E-06	3.33E-014	1.43E-03	1.00E-05	1.78E-011	3.73E-03	4.69E-01	7.0E-019	3.07E-08	2.11E-010	3.74E-016	7.84E-08
Methoxychlor	2.98E-08	4.0E-016	1.91E-05	1.351E-07	2.39E-013	4.99E-05						
Dichlorvos	2.70E-07	4.00E-015	1.74E-04	1.23E-06	2.10E-012	4.52E-04	3.91E-011	5.80E-015	2.52E-08	1.79E-010	3.04E-016	6.55E-08
Malathion	6.36E-010	1.12E-016	4.09E-06	2.89E-08	5.10E-014	1.06E-05	4.87E-014	7.61E-021	3.10E-010	2.19E-012	3.87E-018	8.09E-010
Heptachlor	2.26E-08	4.00E-	1.46E-	1.02E-06	1.82E-	3.80E-	5.08E-	9.00E-	3.28E-	2.31E-09	4.09E-	8.55E-

		015	04		012	04	010	018	07		015	07
Cypermethrin	1.53E-08	2.01E-016	9.85E-06	2.29E-03	1.23E-013	2.56E-05						
Azinphosmethyl	5.95E-010	1.01E-017	3.82E-07	2.69E-09	4.77E-015	9.95E-07						
Tridemorph	1.46E-08	2.01E-016	9.41E-06	6.65E-08	1.17E-014	2.44E-05						
Dimethoate	4.38E-07	5.01E-015	2.81E-04	1.97E-06	3.50E-012	7.30E-04						
Atrazine	3.74E-09	5.71E-017	2.41E-06	1.70E-08	3.01E-014	1.10E-05	2.88E-011	4.40E-019	1.85E-08	1.31E-010	2.31E-016	8.53E-08
Nuarimol	1.41E-08	1.66E-016	9.08E-06	6.41E-08	6.38E-08	2.35E-05						

Table 3: Estimation of Chronic daily intake (CDI) and hazard quotient (HQ) for pesticide residues in sediment from Upper stretch.

	Non-carcinogenic						Carcinogenic					
	Adult			Child			Adult			Child		
	Ingestion	Inhalation	Dermal	Ingestion	Inhalation	Dermal	Ingestion	Inhalation	Dermal	Ingestion	Inhalation	Dermal
Malathion	6.55E-009	1.01E-016	4.22E-06	2.98E-08	5.26E-014	1.09E-05	4.97E-013	7.61E-021	3.20E-010	2.27E-012	4.00E-018	8.35E-010
Heptachlor												
Cypermethrin	8.63E-09	1.01E-016	5.54E-06	3.92E-08	1.27E-09	1.44E-05						
Azinphosmethyl	5.75E-010	1.01E-013	3.69E-07	2.61E-09	3.69E-07	9.61E-07						
Tridemorph	9.58E-09	1.01E-016	6.15E-06	4.35E-08	7.67E-014	1.60E-05						
Nuarimol	1.31E-08	1.66E-016	8.20E-06	5.78E-08	1.02E-013	2.13E-05						
Methyl	5.64	8.01	3.6	2.55	4.51	9.4						

parathion	E-07	E-015	2E-04	E-06	E-012	0E-04						

Table 4: Estimation of Chronic daily intake (CDI) and hazard quotient (HQ) for pesticide residues in sediment from lower stretch.

Non-carcinogenic							Carcinogenic					
	Adult			Child			Adult			Child		
	Ingestion	Inhalation	Dermal	Ingestion	Inhalation	Dermal	Ingestion	Inhalation	Dermal	Ingestion	Inhalation	Dermal
Chlordane	2.38E-06	3.33E-014	1.53E-03	1.08E-05	1.91E-011	3.81E-03	5.00E-011	7.0E-019	3.23E-08	2.27E-010	4.02E-016	8.01E-08
Methoxychlor	2.82E-08	4.0E-016	1.81E-05	1.28E-07	2.25E-013	4.70E-05						
Dichlorvos	2.78E-08	4.00E-015	1.02E-04	1.26E-06	2.24E-012	4.66E-04	4.03E-012	5.80E-019	1.48E-08	1.83E-010	3.24E-016	6.75E-08
Malathion	5.95E-09	1.0E-016	3.82E-06	2.69E-08	4.77E-014	9.95E-06	4.52E-013	7.60E-021	2.90E-010	2.04E-012	3.62E-018	7.56E-010
Heptachlor	2.42E-07	4.00E-015	1.56E-04	1.10E-06	1.95E-012	4.06E-04	5.44E-010	9.00E-018	3.51E-07	2.47E-09	4.39E-015	9.13E-07
Cypermethrin	1.31E-08	1.01E-016	8.44E-06	5.96E-09	1.05E-013	2.19E-05						
Azinphosmethyl	6.35E-010	1.01E-017	4.09E-07	2.89E-09	5.09E-014	1.06E-06						
Tridemorph	1.26E-08	2.01E-016	8.09E-06	5.72E-08	1.00E-013	2.10E-05						
Dimethoate	5.65E-07	1.01E-014	3.65E-04	2.58E-06	4.50E-012	9.50E-04						
Nuarimol	1.96E-08	3.33E-016	1.23E-05	8.66E-08	1.53E-013	3.20E-05						
Methyl	4.01	2.88	2.5	1.81	9.98	6.6						

parathio n	E-07	E- 015	6E- 04	E-06	E- 021	8E- 04						

Table 5: Risk assessment of pesticide residues in sediment samples based on toxic units calculation:

Pesticide	Middle stretch	Upper stretch	Lower stretch
Chlordane	1.25E-02		1.10E-02
Methoxychlor	1.39E-02		1.32E-02
Dichlorvos	6.75E-02		6.99E-02
Malathion	2.24E-02	2.62E-02	2.11E-02
Heptachlor	8.99E-03		9.69E-03
Cypermethrin	1.13E-02	7.19E-03	9.74E-03
Azinphosmethyl	1.86E-02	2.03E-02	2.00E-02
Tridemorph	1.79E-02	1.32E-02	1.55E-02
Dimethoate	3.22E-02		4.19E-02
Atrazine	2.14E-02		
Binapacryl	8.52E-03	1.50E-02	1.23E-02
Nuarimol	1.18E-02	1.20E-02	1.60E-02
Methyl parathion		2.41E-02	5.38E-02

Click here to access/download
Supporting Information
Supplimentary.docx

Click here to access/download
Supporting Information
Supplimentary file.docx

Response to the reviewer’s comments.

Reviewer 1:

ABSTRACT:

1. On line 7, the word "the" before pesticides residues and sediments should be deleted.

Response: the word ‘the’ has been deleted before pesticide residues.

2. On line 10, put the word "the" before upper stretch

Response: Word ‘the’ has been put before upper stretch.

3. On line 11, put the word "and" between ug/kg and in the lower stretch

Response: The word ‘and’ has been put between ug/kg and in the lower stretch.

4. On line 17, delete the word "associated" either before or risk

Response: the word ‘associated’ has been deleted after risk.

5. On line 18, insert the word "to" after due

Response: the word ‘to’ have been inserted after due.

6. On line 19, insert a coma after therefore

Response: ‘coma’ has been inserted after ‘therefore’.

7. On line 19, I guess you want to make a recommendation. You could put it like this "the present study recommends concrete efforts to be made by government to reduce the threat

Response: The recommendation line has been reframed accordingly as suggested by the reviewer.

INTRODUCTION

1. On line 23, insert the word "that" before are

Response: word ‘that’ has been inserted before are.

2. On line 29, insert a coma after furthermore

Response: ‘coma’ has been inserted after ‘furthermore’.

3. On line 31, delete the word "the" before organisms

Response: word ‘the’ has been deleted before organism.

4. On line 33, delete the word "the" before pesticides

Response: word ‘the’ has been deleted before ‘pesticide’.

5. On line 39, insert a full stop (.) after aquatic ecosystem and start a new sentence with Therefore

Response: ‘full stop’ has been added after aquatic ecosystem and new sentence has been started with ‘Therefore’.

6. On line 42, delete the word "but" and put a coma after toxicity and insert "these are"

Response: word ‘but’ has been deleted and ‘coma’ has been added after toxicity and these are has been added before rarely applied.

7. On line 50, delete the word "the" before often

Response: word ‘the’ has been deleted before ‘often’.

8. On line 54, delete "therefore emerge as a concern" and replace it with "This raises a serious concern"

Response: the phrase ‘therefore emerge as a concern’ has been replaced with ‘This raises a serious concern’.

9. On line 57, insert a coma after "in addition" and delete the word "the" before human

Response: ‘coma’ has been added after ‘in addition’ and word ‘the’ has been deleted before human.

10. On line 59, replace the word "accessing" with "assessing"

Response: the word ‘accessing’ has been replaced with ‘assessing’.

11. On line 62, replace the word "accessing" with "assessing"

Response: the word ‘accessing’ has been replaced with ‘assessing’.

12. On line 67, delete the word "evaluation" after risk assessment

Response: the word ‘evaluation’ has been deleted after risk assessment.

13. From line 78 to 86, the objective of the study is not well stated. After the first sentence, the rest look more of a methodology. You could open up the first sentence and continue like this, "and based on these concentrations, determine the risk on non target organisms and humans. This should suffice as the objective of the study. The talk about deterministic and probabilistic approaches can be taken to the materials and methods

Response: The objective of the study has been reframed as suggested by the reviewer.

MATERIALS AND METHODS:

1. On line 101 and 102, put Mutua et al., 2015 into brackets

Response: the reference (Mutua *et al.*, 2015) of the study has been placed in the brackets.

2. On line 103, delete letter "a" between round and bottom and place it before round

Response: the letter ‘a’ has been deleted between round and bottom and placed before round.

3. On line 108, replace chromatogram with chromatography

Response: the word ‘chromatogram’ has been corrected to ‘chromatography’.

4. On line 109, delete the phrase "has been" and insert "were"

Response: The phrase ‘has been’ is replaced with ‘were’.

5. On line 123, add s to difference to make it differences

Response: letter ‘s’ has been inserted after ‘difference’.

6. On line 132, delete "have been" and insert "were"

Response: The phrase ‘has been’ is replaced with ‘were’.

7. On line 164, delete "has been" and insert was

Response: The phrase ‘has been’ is replaced with ‘was’.

8. On line 167, replace slop with slope

Response: word ‘slop’ is replaced with ‘slope’.

RESULTS AND DISCUSSION:

1. On line 181, replace "system that drains" with "systems that drain"

Response: Phrase "system that drains" has been replaced with "systems that drain".

2. On line 182, "Classes of pesticides" and not "class of pesticides"

Response: Phrase ‘class of pesticides’ has been replaced with ‘classes of pesticides’.

3. On line 214, gases that "affect" and not "affects"

Response: Word ‘affects’ has been replaced with ‘affect’.

4. On line 216, insert the word "that" after indicates

Response: word ‘that’ has been inserted after indicates.

5. On line 217, put figure 4 into brackets

Response: ‘figure 4’ has been placed in brackets.

6. On line 218, "three stations" and not "three station"

Response: Phrase ‘three station’ has been replaced with ‘three stations’.

7. On line 221, the word "Thus" should be written with "t"

Response: The word ‘Thus’ has been replaced with ‘thus’.

8. From line 230 to 232, something is missing in the sentence. Could it read as "the higher the organic matter content, the higher the partition coefficient?

Response: The sentence has been corrected as suggested by the reviewer.

9. On line 231, "compounds get" and not "compounds gets"

Response: The word ‘compounds gets’ has been replaced with ‘compounds get’.

10. On line 250, insert a semi colon (;) after three routes

Response: ‘semi colon’ has been inserted after three routes.

11. On line 251, "three stations" and not "three station"

Response: the word ‘three station’ has been replaced with ‘three stations’

12. On line 253, insert a coma after US EPA guidelines

Response: ‘coma’ has been inserted after US EPA guidelines.

CONCLUSION

1. On line 275, delete the word "the" after provide

Response: word ‘the’ has been deleted after provide.

Reviewer 2

1. As authors are dealing with sediments it would be interesting to know the depth of sampling. Please provide it.

Response: The samples used in the study were bottom sediments as explained in line number 99 and 100.

2. Materials and methods section should be improved. Nothing is said about the Chemical and Reagents used. The clean-up must be explained. The quality control is poorly explained. Nothing is said about LOD, LOQs etc. Recoveries should be included together with LODs and LOQs in a table.

Response: Reagents and chemicals used in the experiment were HPLC as mentioned in line number 120, the recovery rate are generally mentioned in line number 123 and ranged in 75-90% as mentioned. The solvent used for extraction were hexane and acetone (1:1) as mentioned in line number 108, the clean-up of extract is explained in section Extraction of samples line 108-110. The LOD and LOQ are not mentioned in this paper as were mentioned in our earlier research paper (https://www.researchgate.net/deref/https%3A%2F%2Fdoi.org%2F10.1016%2Fj.toxrep.2021.08.013) and due to limitations could not be placed in this manuscript.

3. All abbreviations and variables used through the text and in the equations must be explained when used.

Response: The abbreviations and variables used in the text are check again further corrected and explained as suggested by the reviewer.

4. There are some old references (1997) that probably can be updated.

Response: The reference 1997 in line number 163 used is the standard reference from USEPA thus could not be replaced until new standards are not developed.

5. Discussion of PCA results tends to be poor. Authors are suggested to take advantage of these results to explain possible similar sources among other information that can be extracted from PCA.

Response: The PCA part line number 245-253 has been discussed well again with proper suggestions from supervisor and explained well according to the results obtained as suggested by the reviewer.

6. There are some contradictions about human and ecosystem health risk results in the Conclusions section that should be revised.

Response: The human and ecosystem health risk are obtained using different formula given by USEPA. The human health risk assessment results are obtained by using formula for three exposure routes inhalation, dermal and ingestion in adults and children groups of population. While, ecological risks are associated with organisms inhabiting the aquatic ecosystem and calculated by toxic units. Thus both associated risk results are supposed to be different according to the formula used. Further the section was well revised by supervisor and expert in the field.

7. Figures 1-3 should be revised.

Response: The whisker's plots are revised and plotted again as suggested by the reviewers represent number of samples analysed for the study along with obtained mean values, standard error, standard deviation and extremes. The concentration is given in µg/kg values.

Limit of detection and limit of quantification are included in supplementary part as (Table S2)

Line number 289 the toxic unit values are calculated to find out effect of pesticides on algae, daphnia and fish of ecosystem. Whereas line number 283 and 286 sums up impact of pesticide concentration on humans associated to ecosystem. For conformation please check Human risk estimation and Toxic units in methodology part.

Table S3 the maximum and mean value for ingestion rate and inhalation rate has been taken from USEPA set benchmark data.

Study of water for presence of pesticides is included in our first paper accepted in Toxicology reports. Shah, Z.U. and Parveen, S., 2021. Pesticide residues in Rita rita and Cyprinus carpio from river Ganga, India, and assessment of human health risk. *Toxicology Reports*, *8*, pp.1638-1644.
