# Supplementary material for: Distribution and risk assessment of pesticide residues in sediment samples from river Ganga, India
Source: PLoS One. 2023 Feb 2;18(2):e0279993. doi: 10.1371/journal.pone.0279993 (PMC9894440; doi:10.1371/journal.pone.0279993)
Supplement: Attachment — Submitted filename: Response to reviewers.docx [file pone.0279993.s006.docx]

**Response to the reviewer’s comments.**

**Reviewer 1:**

**ABSTRACT:**

1. On line 7, the word "the" before pesticides residues and sediments should be deleted.

**Response:** the word ‘the’ has been deleted before pesticide residues.

2. On line 10, put the word "the" before upper stretch

**Response:** Word ‘the’ has been put before upper stretch.

3. On line 11, put the word "and" between ug/kg and in the lower stretch

**Response:** The word ‘and’ has been put between ug/kg and in the lower stretch.

4. On line 17, delete the word "associated" either before or risk

**Response:** the word ‘associated’ has been deleted after risk.

5. On line 18, insert the word "to" after due

**Response:** the word ‘to’ have been inserted after due.

6. On line 19, insert a coma after therefore

**Response:** ‘coma’ has been inserted after ‘therefore’.

7. On line 19, I guess you want to make a recommendation. You could put it like this "the present study recommends concrete efforts to be made by government to reduce the threat

**Response:** The recommendation line has been reframed accordingly as suggested by the reviewer.

**INTRODUCTION**
1. On line 23, insert the word "that" before are

**Response:** word ‘that’ has been inserted before are.

2. On line 29, insert a coma after furthermore

**Response:** ‘coma’ has been inserted after ‘furthermore’.

3. On line 31, delete the word "the" before organisms

**Response:** word ‘the’ has been deleted before organism.

4. On line 33, delete the word "the" before pesticides

**Response:** word ‘the’ has been deleted before ‘pesticide’.

5. On line 39, insert a full stop (.) after aquatic ecosystem and start a new sentence with Therefore

**Response:** ‘full stop’ has been added after aquatic ecosystem and new sentence has been started with ‘Therefore’.

6. On line 42, delete the word "but" and put a coma after toxicity and insert "these are"

**Response:** word ‘but’ has been deleted and ‘coma’ has been added after toxicity and these are has been added before rarely applied.

7. On line 50, delete the word "the" before often

**Response:** word ‘the’ has been deleted before ‘often’.

8. On line 54, delete "therefore emerge as a concern" and replace it with "This raises a serious concern"

**Response:** the phrase ‘therefore emerge as a concern’ has been replaced with ‘This raises a serious concern’.

9. On line 57, insert a coma after "in addition" and delete the word "the" before human

**Response:** ‘coma’ has been added after ‘in addition’ and word ‘the’ has been deleted before human.

10. On line 59, replace the word "accessing" with "assessing"

**Response:** the word ‘accessing’ has been replaced with ‘assessing’.

11. On line 62, replace the word "accessing" with "assessing"

**Response:** the word ‘accessing’ has been replaced with ‘assessing’.

12. On line 67, delete the word "evaluation" after risk assessment

**Response:** the word ‘evaluation’ has been deleted after risk assessment.

13. From line 78 to 86, the objective of the study is not well stated. After the first sentence, the rest look more of a methodology. You could open up the first sentence and continue like this, "and based on these concentrations, determine the risk on non target organisms and humans. This should suffice as the objective of the study. The talk about deterministic and probabilistic approaches can be taken to the materials and methods

**Response:** The objective of the study has been reframed as suggested by the reviewer.

**MATERIALS AND METHODS:**

1. On line 101 and 102, put Mutua et al., 2015 into brackets

**Response:** the reference (Mutua *et al*., 2015) of the study has been placed in the brackets.

2. On line 103, delete letter "a" between round and bottom and place it before round

**Response:** the letter ‘a’ has been deleted between round and bottom and placed before round.

3. On line 108, replace chromatogram with chromatography

**Response:** the word ‘chromatogram’ has been corrected to ‘chromatography’.

4. On line 109, delete the phrase "has been" and insert "were"

**Response:** The phrase ‘has been’ is replaced with ‘were’.

5. On line 123, add s to difference to make it differences

**Response:** letter ‘s’ has been inserted after ‘difference’.

6. On line 132, delete "have been" and insert "were"

**Response:** The phrase ‘has been’ is replaced with ‘were’.

7. On line 164, delete "has been" and insert was

**Response:** The phrase ‘has been’ is replaced with ‘was’.

8. On line 167, replace slop with slope

**Response:** word ‘slop’ is replaced with ‘slope’.

**RESULTS AND DISCUSSION:**

1. On line 181, replace "system that drains" with "systems that drain"

**Response:** Phrase "system that drains" has been replaced with "systems that drain".

2. On line 182, "Classes of pesticides" and not "class of pesticides"

**Response:** Phrase ‘class of pesticides’ has been replaced with ‘classes of pesticides’.

3. On line 214, gases that "affect" and not "affects"

**Response:** Word ‘affects’ has been replaced with ‘affect’.

4. On line 216, insert the word "that" after indicates

**Response:** word ‘that’ has been inserted after indicates.

5. On line 217, put figure 4 into brackets

**Response:** ‘figure 4’ has been placed in brackets.

6. On line 218, "three stations" and not "three station"

**Response:** Phrase ‘three station’ has been replaced with ‘three stations’.

7. On line 221, the word "Thus" should be written with "t"

**Response:** The word ‘Thus’ has been replaced with ‘thus’.

8. From line 230 to 232, something is missing in the sentence. Could it read as "the higher the organic matter content, the higher the partition coefficient?

**Response:** The sentence has been corrected as suggested by the reviewer.

9. On line 231, "compounds get" and not "compounds gets"

**Response:** The word ‘compounds gets’ has been replaced with ‘compounds get’.

10. On line 250, insert a semi colon (;) after three routes

**Response:** ‘semi colon’ has been inserted after three routes.

11. On line 251, "three stations" and not "three station"

**Response:** the word ‘three station’ has been replaced with ‘three stations’

12. On line 253, insert a coma after US EPA guidelines

**Response:** ‘coma’ has been inserted after US EPA guidelines.

**CONCLUSION**
1. On line 275, delete the word "the" after provide

**Response:** word ‘the’ has been deleted after provide.

**Reviewer 2**

1. As authors are dealing with sediments it would be interesting to know the depth of sampling. Please provide it.

**Response:** The samples used in the study were bottom sediments as explained in line number 99 and 100.

2. Materials and methods section should be improved. Nothing is said about the Chemical and Reagents used. The clean-up must be explained. The quality control is poorly explained. Nothing is said about LOD, LOQs etc. Recoveries should be included together with LODs and LOQs in a table.

**Response:** Reagents and chemicals used in the experiment were HPLC as mentioned in line number 120, the recovery rate are generally mentioned in line number 123 and ranged in 75-90% as mentioned. The solvent used for extraction were hexane and acetone (1:1) as mentioned in line number 108, the clean-up of extract is explained in section Extraction of samples line 108-110. The LOD and LOQ are not mentioned in this paper as were mentioned in our earlier research paper (<https://www.researchgate.net/deref/https%3A%2F%2Fdoi.org%2F10.1016%2Fj.toxrep.2021.08.013>) and due to limitations could not be placed in this manuscript.

3. All abbreviations and variables used through the text and in the equations must be explained when used.

**Response:** The abbreviations and variables used in the text are check again further corrected and explained as suggested by the reviewer.

4. There are some old references (1997) that probably can be updated.

**Response:** The reference 1997 in line number 163 used is the standard reference from USEPA thus could not be replaced until new standards are not developed.

5. Discussion of PCA results tends to be poor. Authors are suggested to take advantage of these results to explain possible similar sources among other information that can be extracted from PCA.

**Response:** The PCA part line number 245-253 has been discussed well again with proper suggestions from supervisor and explained well according to the results obtained as suggested by the reviewer.

6. There are some contradictions about human and ecosystem health risk results in the Conclusions section that should be revised.

**Response:** The human and ecosystem health risk are obtained using different formula given by USEPA. The human health risk assessment results are obtained by using formula for three exposure routes inhalation, dermal and ingestion in adults and children groups of population. While, ecological risks are associated with organisms inhabiting the aquatic ecosystem and calculated by toxic units. Thus both associated risk results are supposed to be different according to the formula used. Further the section was well revised by supervisor and expert in the field.

7. Figures 1-3 should be revised.

**Response:** The whisker’s plots are revised and plotted again as suggested by the reviewers represent number of samples analysed for the study along with obtained mean values, standard error, standard deviation and extremes. The concentration is given in µg/kg values.

**Limit of detection and limit of quantification** are included in supplementary part as (Table S2)

**Line number 289** the toxic unit values are calculated to find out effect of pesticides on algae, daphnia and fish of ecosystem. Whereas line number 283 and 286 sums up impact of pesticide concentration on humans associated to ecosystem. For conformation please check Human risk estimation and Toxic units in methodology part.

**Table S3** the maximum and mean value for ingestion rate and inhalation rate has been taken from USEPA set benchmark data.

**Study of water** for presence of pesticides is included in our first paper accepted in Toxicology reports. Shah, Z.U. and Parveen, S., 2021. Pesticide residues in Rita rita and Cyprinus carpio from river Ganga, India, and assessment of human health risk. *Toxicology Reports*, *8*, pp.1638-1644.
