# Supplementary material for: Distribution and risk assessment of pesticide residues in sediment samples from river Ganga, India
Source: PLoS One. 2023 Feb 2;18(2):e0279993. doi: 10.1371/journal.pone.0279993 (PMC9894440; doi:10.1371/journal.pone.0279993)
Supplement: Attachment — Submitted filename: Response to the reviewers second revision.docx [file pone.0279993.s008.docx]

**Response to the reviewer’s comments:**

**Editor Comment:** This study must give convinced reasons why only pesticides in sediment were investigated, without consideration of their levels and risk in water.

**Response:** In our early research paper we have detected the level of pesticides in water from the studied area and there is lot of literature available on pesticide presence in water the river Ganga. Our main focus was to find out concentration of pesticides in sediment samples from the studied river and determination of probabilistic risk posed by the pesticides by calculating human and ecological risk. Few of the papers are mentioned below that have observed pesticides in water from river Ganga.

Divya, R., Ruby, P., Vikash, P., Sharma, P.K. and Shukla, D.N., 2014. Physico-chemical and pesticide analysis of River Ganga in Allahabad City, Uttar Pradesh, India. *Asian Journal of Biochemical and Pharmaceutical Research*, *4*(3), pp.239-244.

Mutiyar, P.K. and Mittal, A.K., 2013. Status of organochlorine pesticides in Ganga river basin: anthropogenic or glacial?. *Drinking Water Engineering and Science*, *6*(2), pp.69-80.

Leena, S., Choudhary, S.K. and Singh, P.K., 2011. Organochlorine and organophosphorous pesticides residues in water of River Gangaat Bhagalpur, Bihar, India. *Int J Res Chem Environ*, *1*, pp.77-84.

Singh, L., Choudhary, S.K. and Singh, P.K., 2012. Pesticide concentration in water and sediment of River Ganga at selected sites in middle Ganga plain. *International journal of environmental sciences*, *3*(1), pp.260-274.

Shah, Z.U. and Parveen, S., 2021. Pesticide residues in Rita rita and Cyprinus carpio from river Ganga, India, and assessment of human health risk. *Toxicology Reports*, *8*, pp.1638-1644.

Samanta, S., 2013. Metal and pesticide pollution scenario in Ganga River system. *Aquatic ecosystem health & management*, *16*(4), pp.454-464.

**Editor comment:** We note that figure 1 in your submission contain [map/satellite] images which may be copyrighted.

**Response:** Figure 1 has been developed by the author by using software as mentioned in the caption of figure. There is no copyright issue with the figure to the best of our knowledge. Further if the editor finds any copyright issue please highlight the source so the authors could right for permission to the actual author.

**Reviewer 1:**

1. Several suggestions made by the reviewers that are not included in the text (for instance there is still a lack of information and explanations requested several times as the SPE, LOD, LOQs, etc.

**Response:** SPE along with its methodology has been explained well in the section extraction of samples line number 112- 122. LOD, LOQ and recovery percentage of the detected pesticides has been well documented in the supplementary part (Table S2). The sediment samples were fortified with 10 µg/kg of the pesticides in sediments. Please refer to the supplementary part table S1.

2. Supplementary Information is not explained and there are data missing.

**Response:** In the supplementary part Table S1 gives few international studies on pesticide concentration detected in sediment samples as is explained in the manuscript line number 277-284, Table S2 gives the LOD, LOQ and Recovery percentage of each studied pesticide and is well explained in the quality control and quality assurance section line number 141-146, Table S3 in the supplementary file depicts input data from USEPA for the calculation of risk assessment, Table S4 gives the oral reference dose and cancer slop factor of each studied pesticides used for calculation of probable human health risk.

3. Table 2, Figures 2, 3, 4 and lines 199-213 present the same information.

**Response:** Table 1 has been removed as figure 2, 3 and 4 present the same data as suggested by reviewer. Line number 199 – 213 explains the data well as it could not be properly explained in the figures.

4. Higher amounts of TOC were mentioned in middle and lower stretches, thus pesticide deposition was higher than upper stretch.

**Response:** No statistical support is present in this section; **Total Organic Carbon** was determined by partial oxidation method following Walkey and Black method as described by Trivedy and Goel (1998).

**Reviewer 2:**

1. Aspects related to the risk posed by pesticides are not properly presented.

**Response:** The section has been included in the introduction part in first paragraph line number 37-44.

2. The scope of the paper is not defined and the human health risk due to exposure from contaminated sediment is not justified.

**Response:** Line number 86-89, 306-319, 313-315, 31-40, 280-294 mainly focus on scope and human health risk due to exposure of contaminated sediments.

3. The description of the analysis is not clear. The authors reported the use of LC-MS system for the detection of pesticide residues and the use of a GC column and ECD detection!!! Moreover, the operational conditions are not presented.

**Response:** the section has been reframed and operational conditions have been included in the section.

4. It would be interesting to include a discussion section with data of currently used pesticides in the studied area and the legacy pesticides that have been detected.

**Response:** The section has been included in discussion part line number 243-250.
